# Strategizing Earth Science Data Development

**DOI:** 10.1038/s41597-024-03531-6

**Published:** 2024-06-26

**Authors:** Zhong Liu, Tian Yao

**Affiliations:** 1 George Mason University and NASA Goddard Earth Sciences (GES) Data and Information Services Center (DISC), Greenbelt, Maryland USA; 2 Science Systems and Applications Inc. and NASA GSFC Terrestrial Information Systems Laboratory, Greenbelt, Maryland USA

**Keywords:** Environmental sciences, Scientific community

## Abstract

Developing Earth science data products that meet the needs of diverse users is a challenging task for both data producers and service providers, as user requirements can vary significantly and evolve over time. In this comment, we discuss several strategies to improve Earth science data products that everyone can use.

## Introduction

Emerging technologies such as artificial intelligence (AI), machine learning (ML), and cloud computing are making Earth science data an increasingly valuable resource for research, applications, and education. Producing Earth science data everyone can use will enhance data-driven scientific research, develop effective action plans for mitigating climate change and natural disasters, maximize investment in global observations, research, and modeling efforts, and educate the next generation to be well-prepared for environmental changes.

User needs for Earth science data can vary significantly and evolve over time. For example, weather forecasters rely on low-latency observations and model data. Climate scientists seek long-term, high-quality datasets. Although most Earth science data products are openly available and searchable, only a few, if they exist, are close to or meet user needs. To understand these specific requirements, the National Aeronautics and Space Administration (NASA) has annually conducted the American Customer Satisfaction Index (ACSI) survey^1^ of users of NASA’s Earth Observing System Data and Information System (EOSDIS) Distributed Active Archive Centers (DAACs)^2^ since 2004. Survey results indicate that non-professional users face greater challenges discovering NASA Earth data than professionals, such as university professors. The 2022 community assessment report^3^ for NASA’s Atmosphere Observing System (AOS) mission highlights varied user needs across disciplines, including latency, spatiotemporal resolution, data coverage, and data continuity. With global warming and more frequent hazard events, the demand for data to train AI/ML models and inform decision-making has surged, bringing a significant challenge to developing Earth science data products that meet user needs.

The data product development lifecycle includes the analysis of user needs, data collection, product development and processing, production dissemination, and metrics. There are many articles and discussions about the Earth system user needs, products, data services, data sharing, standards, and the FAIR (findable, accessible, interoperability, and reusable) principles^4–13^. However, discussions about solutions and strategies to systematically improve data products for a broader user community are limited. Based on previous studies^5^, we outline strategies for each component of the data product development lifecycle below.

## User Needs

User needs are paramount because, ultimately, it is the user who decides if data products are appropriate for their needs, which are diverse. User needs are also closely related to all four areas of the FAIR guiding principles^4^ and more (e.g., data quality, data ethics, and latency).

Findability^4,7^ is arguably the most important, but also challenging. Users mostly depend on search engines to find data. Because there are so many data repositories around the world, developing standardized data catalogs for all data is imperative, requiring cross-organizational and international collaboration. One obstacle is the development of standardized data products with metadata that are FAIR-compliant, which can be costly (e.g., reprocessing existing products for compliance) and involve a culture challenge (e.g., additional work and cost for product development).

In the current environment, users are dependent on the data products that are available. In many cases, data products need to be customized by them to meet their individual needs. In theory, user needs should be analyzed first to guide strategies for data collection, product development, and product dissemination. However, for mainly historical reasons, this is often not the case. Data products are typically generated by principal investigators or teams for specific missions or projects. When a project ends, it is very likely that their product development and maintenance activities will stop as well. Without continuous support and sustainable long-term plans, the usefulness of such a product can be limited. As an example, if product updates cease, activities and services that are dependent upon the product may cease as well.

In short, collecting and analyzing user needs should be integrated into strategies for data product development.

## Data Collection

Over the past several decades, advancements in space-borne, air-borne, and ground-based remote sensing instruments have significantly expanded and enhanced the collection of Earth observations. Despite many efforts to collect data, gaps in observations still exist, especially over vast oceans and remote continental areas.

Data gaps can influence data quality, which impacts a wide range of activities such as continuously monitoring Earth’s conditions and studying climate and disaster events. It can be challenging to develop high spatiotemporal resolution products that many users need for local and regional applications due to gaps in the data. Data gaps are often filled with products of mixed quality when there are insufficient global or regional observation networks. It is also not easy to provide data quality information for such integrated data products. Further research activities are needed to better communicate with users, such as providing data quality information and guidelines for using data products properly.

**Fig. 1:**
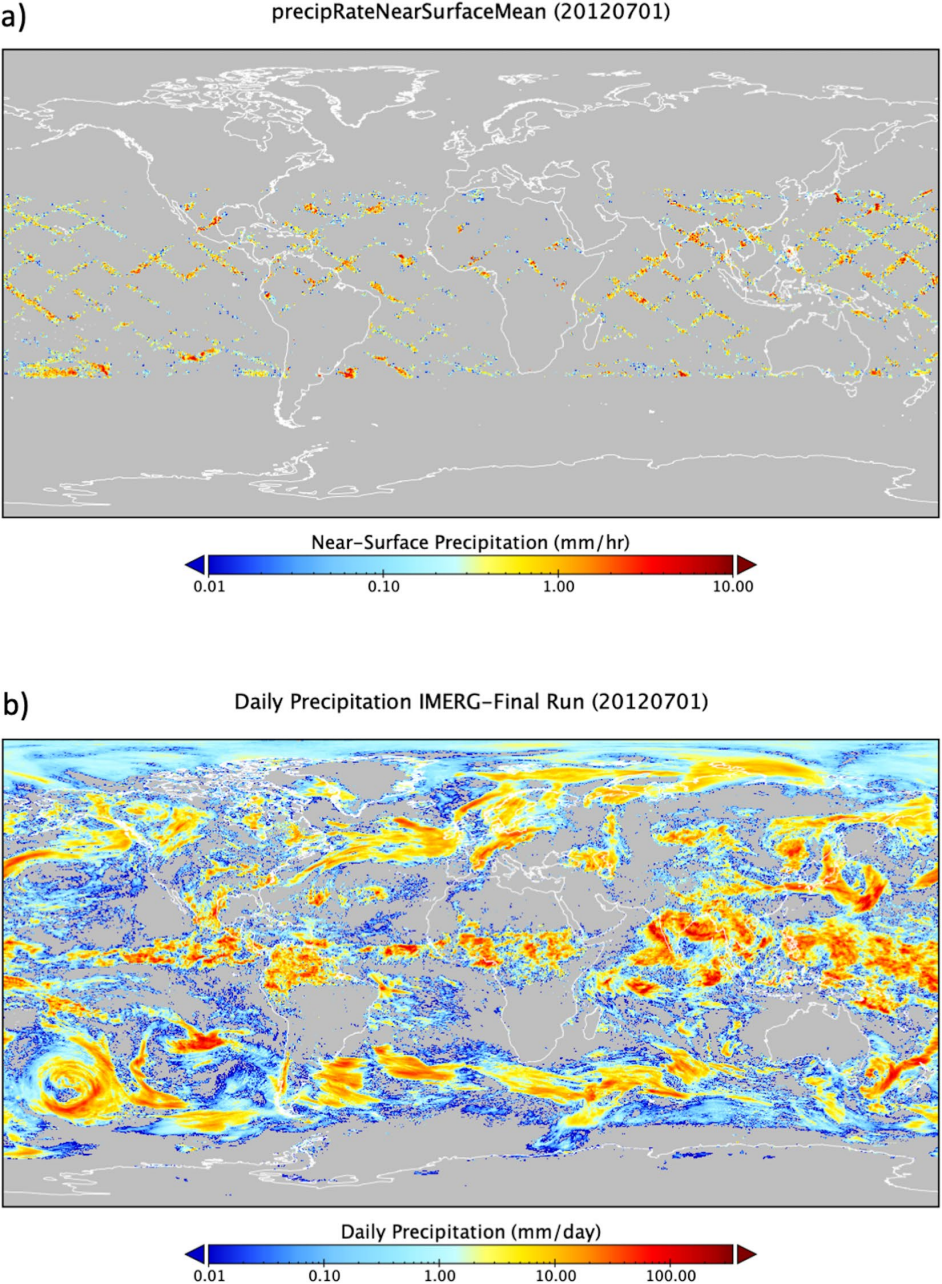
Global daily precipitation maps on July 1, 2012: (**a**) Mosaic of snapshots/orbits of precipitation estimates from the first space-borne Ku-band weather radar onboard the NASA-JAXA TRMM satellite, showing marked data gaps in both space and time with measurements from a single satellite; and (**b**) Daily accumulation of the Integrated Multi-satellitE Retrievals for GPM (IMERG) product utilizing a constellation of international satellites with calibration from ground-based observations, improving data availability significantly. However, the data quality of each pixel may vary.

Figure 1a, for example, shows a daily orbital mosaic^14^ of the first space-borne weather radar (Ku-band) onboard the Tropical Rainfall Measuring Mission^15^ (TRMM), a joint satellite mission launched in 1997 by NASA and the Japanese Aerospace Exploration Agency^16^ (JAXA). As seen, large data gaps exist in space and time (Fig. 1a). To provide continuous monitoring of global precipitation, data gaps in observations are currently filled from a constellation of international satellites. However, the quality of satellite sensors (e.g., passive microwave and infrared sensors) in this constellation varies, and the resulting data products can be affected. Figure 1b is the daily global precipitation map at 0.1 degree x 0.1 degree grid resolution on the same date as in Fig. 1a, generated from NASA’s Global Precipitation Measurement^17^ (GPM) mission precipitation product^18^, the Integrated Multi-satellitE Retrievals for GPM (IMERG). The IMERG product contains far fewer data gaps compared to the radar product.

Data quality information, including consistency, continuity, uncertainty, bias, latency, and resolution, is a major concern for scientific and application users. Requirements for quality information also vary between user communities. For example, real-time data may not have the same quality as their climate data records, which are well calibrated and consistent. It is difficult for scientists to develop specific products for each research or application scenario. Strategies that utilize data services will be described in Product Dissemination.

Over the years, Earth observations have been carried out by different organizations (e.g., NASA, NOAA, USGS) and activities (e.g., field campaigns), and more observations will be available through new missions, such as the NASA Earth System Observatory^19^. In recent years, commercial companies have increasingly played an important role in Earth observation activities. Without a platform and standards, sharing data to fill existing data gaps and making data FAIR-compliant are difficult. Evolving information technologies have enabled scientific communities to become more and more interactive and collaborative through Internet-based platforms^6^ to break data and knowledge silos and organizational boundaries. As data needs increase, it has become more urgent to support and explore various solutions or open data-sharing platforms by integrating data from government, private, and non-government sources, such as a proposed consortium solution^6^ supported by stakeholders. On the other hand, planning future satellite missions requires a balance between filling observational data gaps and experimenting with new observation methods to improve scientific knowledge and data quality.

## Product Development

In theory, ideal data products consist of long-term, global, well-calibrated, consistent, bias-free, low-uncertainty, low-latency data records at fine spatial and temporal scales. Realistically, such products are very limited and difficult to locate.

Strategies need to be developed for continuously improving data products. First, data product availability and quality improvement require a long-term, sustained commitment. Otherwise, it can be difficult for users, especially operational users, to plan, use, and depend on data products in their activities. Dedicated resources must be available to support a product’s life cycle. NASA’s Making Earth System Data Records for Use in Research Environments^20^ (MEaSUREs) program is an example of producing climate data records through data integration. However, when a project ends, product support and updates often end as well. Essential climate variables^21^ proposed by the United Nations World Meteorological Organization (WMO) are increasingly being used across a diverse range of domains and disciplines, especially in the environmental sciences, which could be the starting point for producing data products through systematic data integration.

Data assimilation products will increasingly play a critical role in providing Earth’s information. Data assimilation uses a technique to analyze the state of the Earth by combining model data and observations. For example, numerical-model-based precipitation estimates (e.g., NASA’s Modern-Era Retrospective analysis for Research and Applications, Version 2 (MERRA-2)^22^) may have the potential to add value in high latitudes due to shortcomings in microwave measurement^23^. Combining both models and observations may generate higher quality global precipitation products. However, data assimilation products still need improvements (e.g., spatiotemporal resolution) to meet the requirements of the ideal products mentioned earlier.

Implementing these new strategies can be a challenge because data producers need to address all data-related issues, such as quality, consistency, and long-term availability. But fewer, higher-quality data products will benefit users who may have a hard time finding a suitable product among many similar products, encouraging long-term commitment.

For diverse user communities, their product needs can vary significantly. For example, some seek daily products, while others seek 10-day products. A strategy is for data producers to focus on the development of their ideal data products and rely on data service providers to generate customized data products for specific user communities.

## Product Dissemination

Product dissemination plays a key role in research, applications, and data democratization. Without proper product dissemination, the best products may have difficulty reaching their potential users and therefore limit their usefulness and impact. For example, the most recent NASA “Earth Science to Action Strategy 2024–2034”^24^ outlines that the Earth Information Center (EIC) is designated to function as a unified portal, facilitating access to data, information, tools, and solutions to support a diverse range of users, stakeholders, decision-makers, policymakers, and the public. There are many challenges to product dissemination, such as making data FAIR and open^25^.

One strategy is to develop more FAIR-compliant, value-added, processing-oriented data services to facilitate data access and exploration (e.g., NASA’s Giovanni^26^) as well as reduce data egress and related costs. These services can produce customized data products that are not in data repositories. For example, the daily IMERG product (Fig. 1b) is generated from the original half-hourly product that is sent to the archive center. Customized products, including data quality, can be a part of analysis ready data (ARD) development to facilitate data analysis and increase scientific productivity. For instance, the Committee on Earth Observation Satellites (CEOS) has established a CEOS-ARD framework and strategy^27,28^. The CEOS Land Surface Information Virtual Constellation (LSI-VC) has been leading the CEOS Analysis Ready Data (CARD4L) initiative for a few years. To date, the U.S. Geological Survey (USGS) Landsat Collection 2 has been processed and has a CEOS ARD seal of approval and recognition. ARD services are also needed for non-satellite products (e.g., in-situ observations, model data). The involvement of data producers is a must to ensure that data processing is handled properly.

Data dissemination can be further expanded for users with different backgrounds. Based on user needs, analytical functions and visualizations can be developed to facilitate data exploration, scientific discovery, learning, and outreach activities. Different tools need to be provided by data service providers to disseminate data and information to different users. For example, ordinary people often use smartphone apps (e.g., weather apps) to obtain Earth’s environmental information. Data service providers need to provide such apps accordingly.

## Metrics

Metrics^29^ are essential for monitoring, analyzing, and benchmarking all data and service-related activities, ranging from problems that users encounter to service operation, collection, data product quality, and FAIR compliance (e.g., NASA’s ACSI survey).

There are several strategies to improve product development, including plans to: 1) identify relevant parameters for collecting metrics in all areas of the product life cycle; 2) collect metrics (e.g., FAIR-compliant); and 3) develop holistic analysis methods. Metrics evolve over time; therefore, adjustments or new metrics need to be considered. An example is the development of metrics for interdisciplinary data and services^29^.

Most metrics are for internal consumption, but they are also increasingly being used in research and other applications. For instance, publishers include metrics (e.g., citations, downloads) in their published articles. By publishing the metrics, users can assess the suitability of a particular dataset or product for their specific application, and data producers can evaluate the usage and impact of a particular data product or service.

## Implementation

We have presented several strategies for improving the development processes of Earth data products to broaden their use for supporting a wide range of activities. Now, the question is: how do we implement these strategies?

In the short term, efforts should be focused on the adoption of best practices and community standards to update or develop data products that are FAIR-compliant. For example, new requirements can be incorporated into data management plans in a research proposal, which has been implemented by some U.S. federal agencies, such as NASA.

For long-term solutions, next-generation infrastructures for Earth science data and computing are needed to allow these strategies to work seamlessly. The infrastructures include data collection, data archives, data services, and scientific computing. For example, NASA’s Earth observations and model data are being migrated to NASA’s Earth data cloud environment^30^, an important component of such infrastructures. Once the migration finishes, datasets at all DAACs will be archived and distributed in one place, the Earthdata cloud^31^, to facilitate new product development and other activities (e.g., interdisciplinary science, applications, and education). The migration will also enable computing close to data, a key improvement for a range of activities such as data product development, validation, and data services down the road.
